# Phase I trial to investigate the effect of renal impairment on isavuconazole pharmacokinetics

**DOI:** 10.1007/s00228-017-2213-7

**Published:** 2017-03-07

**Authors:** Robert W. Townsend, Shahzad Akhtar, Harry Alcorn, Jolene K. Berg, Donna L. Kowalski, Salim Mujais, Amit V. Desai

**Affiliations:** 1 Global Clinical Pharmacology and Exploratory Development Science, Astellas Pharma Global Development, Inc., 1 Astellas Way, Northbrook, IL 60062 USA; 2 DaVita Clinical Research, Minneapolis, MN USA

**Keywords:** End-stage renal disease, Isavuconazole, Pharmacokinetics, Renal impairment

## Abstract

**Purpose:**

The purpose of the study is to evaluate the effect of renal impairment (RI) and end-stage renal disease (ESRD) on the pharmacokinetics (PK) of isavuconazole and the inactive cleavage product, BAL8728.

**Methods:**

A single intravenous dose of the prodrug isavuconazonium sulfate (372 mg, equivalent to 200 mg isavuconazole and 75 mg of BAL8728 cleavage product) was administered to healthy controls (parts 1 and 2) and participants with mild, moderate, or severe RI (part 2) or ESRD (part 1); ESRD participants received two doses of 200 mg isavuconazole, 1 h post-dialysis (day 1) and prior to dialysis (day 15). Plasma PK parameters for isavuconazole included maximum concentration (*C*_max_), area under the concentration–time curve (AUC) from time of dose to 72 h (AUC_72_), AUC extrapolated to infinity (AUC_∞_), AUC to last measurable concentration (AUC_last_), half-life (*t*_½_ h), volume of distribution (*V*_z_), and total clearance (CL), for the healthy control group versus those with mild, moderate, or severe RI or ESRD.

**Results:**

Isavuconazole *C*_max_ values were 4% higher in mild RI and 7, 14, and 21% lower in participants with moderate RI, severe RI, or ESRD versus the healthy control group, respectively. When hemodialysis occurred post-dose (day 15), participants with ESRD had a 30% increase in AUC_72_ for isavuconazole in parallel with reduction of extracellular volume induced by dialysis. Exposure (AUC_∞_ and AUC_last_) was not significantly different for participants with mild, moderate, or severe RI versus healthy controls although there was considerable variability. The t_1/2_ (day 1) was 125.5 ± 63.6 h (healthy control group), 204.5 ± 82.6 h (ESRD group) in part 1, and 140.5 ± 77.7 h (healthy control group), 117.0 ± 66.2 h (mild RI), 158.5 ± 56.4 h (moderate RI), and 145.8 ± 65.8 L/h (severe RI) in part 2. CL was 2.4 ± 0.8 L/h (healthy control group) and 2.9 ± 1.3 L/h (ESRD group) in part 1 and 2.4 ± 1.2 L/h (healthy control group), 2.5 ± 1.0 L/h (mild RI), 2.2 ± 0.8 L/h (moderate RI), and 2.4 ± 0.8 L/h (severe RI) in part 2. The *V*_z_ was 382.6 ± 150.6 L in the healthy control group and 735.6 ± 277.3 L in ESRD patients on day 1 in part 1 of the study. In part 2 of the study, *V*_z_ was 410.8 ± 89.7 L in the healthy control group, 341.6 ± 72.3 L in mild RI, 509.1 ± 262.2 L in moderate RI, and 439.4 L in severe RI.

**Conclusions:**

Based on the findings of this study, dose adjustments of isavuconazole are unlikely to be required in individuals with RI or in those with ESRD who receive hemodialysis.

**Electronic supplementary material:**

The online version of this article (doi:10.1007/s00228-017-2213-7) contains supplementary material, which is available to authorized users.

## Introduction

Invasive fungal diseases (IFD), predominantly aspergillosis, are a prevalent cause of morbidity and mortality in immunocompromised patients, such as those with hematological malignancies or those undergoing transplantation [1–4]. Renal impairment (RI) is an independent risk factor for mortality in both hematopoietic stem cell transplant and solid organ transplant patients with invasive aspergillosis (IA) [5]. In intensive care units, 43% of patients with IA infections experience acute renal failure, which contributes to the mortality associated with IA [6]. The renal excretion of drugs and/or their metabolites may be hindered in patients with RI, and this could lead to an excessive accumulation of the drug in the body [7]. Conversely, hemodialysis may result in removal of some drugs, and thereby, additional doses may be required to prevent underdosing [8]. Triazole antifungal agents are pivotal in the treatment of IA [9]; however, their use may be restricted in patients with RI [10, 11]. Voriconazole and posaconazole may have restricted use in patients with moderate-to-severe RI due to the accumulation of the vehicle cyclodextrin used in their intravenous (IV) formulations [10–12]. Caution is also recommended for the use of itraconazole in patients with RI due to limited data on the use of this drug in this patient population [13]. Therefore, there is a requirement for potent antifungal agents that are efficacious and well tolerated to combat IFD in patients with RI.

Isavuconazonium sulfate is a water-soluble prodrug of the novel, broad-spectrum, triazole antifungal agent isavuconazole, which was developed to facilitate IV administration without the need for nephrotoxic excipients [14, 15]. Isavuconazonium sulfate is rapidly converted in plasma to the active triazole isavuconazole and the inactive cleavage product BAL8728. The per-oral (PO) capsules and cyclodextrin-free IV formulations of the prodrug are approved for the primary treatment of adults with IA and invasive mucormycosis by the US Food and Drug Administration (FDA) [16]. Isavuconazole is also approved by the European Medicines Agency (EMA) for the treatment of IA and treatment of invasive mucormycosis when amphotericin B is inappropriate [17].

A formal renal study using the final formulation, isavuconazonium sulfate, was conducted in accordance with the FDA and the EMA guidance on the evaluation of the pharmacokinetics (PK) of medicines in patients with impaired renal function [18, 19]. The objective of this study was to evaluate the effect of RI (mild, moderate, or severe) and end-stage renal disease (ESRD) on the PK of isavuconazole compared with the PK in healthy participants with normal renal function.

## Methods

### Study design

This was a phase I, open-label, single-dose parallel group study in male and female participants conducted in two parts (ClinicalTrials.gov NCT01555866 covering parts 1 and 2). Part 1 was a single-center study of isavuconazole administered to healthy participants with renal function in the normal range (referred to as the healthy control group) and those with ESRD requiring dialysis. Part 2 was a multi-center study of isavuconazole administered to a healthy control group and those with mild, moderate, or severe RI.

Both parts of the study were conducted in accordance with the Declaration of Helsinki and the International Conference on Harmonisation Guidelines for Good Clinical Practice. For all sites, approval of the protocol (9766-CL-0018) was obtained from the governmental authorities and Institutional Review Board(s).

### Eligibility

Male and female participants aged 18–65 years, weighing ≥45 kg, and with a body mass index of 18–35 kg/m^2^ were enrolled. At screening, RI was based on the Cockcroft–Gault (CG) formula and adjusted for body surface area (BSA), then grouped as healthy control group (creatinine clearance (CL_cr_) >80 mL/min/1.73 m^2^), participants with ESRD and requiring hemodialysis (CL_cr_ < 15 mL/min/1.73 m^2^), and participants with RI: mild (CL_cr_ 50–80 mL/min/1.73 m^2^), moderate (CL_cr_ 30–<50 mL/min/1.73 m^2^), and severe CL_cr_ (<30 mL/min/1.73 m^2^). Participants were selected by age, sex, weight, and smoking status so that the ranges were similar between the healthy control group and each of the groups with RI.

### Assessments

Each participant in part 1 and part 2 of the study received a single 1-h IV infusion of isavuconazonium sulfate 372 mg (equivalent to 200 mg isavuconazole) on day 1 (approximately 1 h after completion of their routine hemodialysis procedure in participants with ESRD). Participants with ESRD in part 1 of the study received an additional dose just prior to dialysis on day 15.

Blood samples for isavuconazole and BAL8728 plasma concentrations were collected pre-dose to 72 h post-dose on days 1 and 15 for ESRD participants and pre-dose to 72 h post-dose on day 1 for the healthy control group and RI participants. Single blood samples were taken from ESRD and RI participants on days 6, 8, 11, 13, and 15. During dialysis, samples were collected simultaneously at the inlet and outlet sides of the dialyzer as well as from the dialysate. For all participants, an additional blood sample was obtained at 4 h post-dose on day 1 for analysis of isavuconazole fraction unbound (fu).

In participants who produced urine, samples for the bioanalysis of isavuconazole and BAL8728 were collected up to 72 h post-dose on day 1. Renal function was assessed using the CG method adjusted for BSA using the following formula:

$$ {\mathrm{CL}}_{\mathrm{cr}}\left(\mathrm{mL}/ \min /1.{73}^2\right)=\frac{\left[140-\mathrm{age}\left(\mathrm{years}\right)\right]\times \mathrm{actual}\kern0.5em \mathrm{weight}\kern0.5em \left(\mathrm{kg}\right)\times 0.85\kern0.5em \mathrm{for}\kern0.5em \mathrm{females}\kern0.5em \times 1.73}{72\times {\mathrm{S}}_{\mathrm{cr}}\left(\mathrm{mg}/\mathrm{dL}\right)\times \mathrm{BSA}} $$

where S_cr_ is serum creatinine.

Estimated glomerular filtration rate (eGFR) using the abbreviated Modification of Diet in Renal Disease (MDRD) formula was calculated using the following formula:

$$ \mathrm{eGFR}-\mathrm{MDRD}\ \mathrm{m}\mathrm{L}/ \min /1.{73\ \mathrm{m}}^2=175\times {{\mathrm{S}}_{\mathrm{cr}}}^{-1.154}\times {\mathrm{age}}^{-0.203}\times \left(0.742\ \mathrm{if}\ \mathrm{female}\right)\times \left(1.212\ \mathrm{if}\ \mathrm{African}\ \mathrm{American}\right) $$

### Pharmacokinetic assessments

Due to the extensive protein binding of isavuconazole to plasma proteins, the PK parameters reported in this study were based on total isavuconazole concentrations in plasma. Plasma PK sampling time points included pre-dose (prior to start of infusion), upon completion of infusion (obtained 1 min prior to end of infusion), 1.5, 2, 3, 4, 5, 6, 8, 12, 24 (day 2), 36 (day 2), 48 (day 3), 72 (day 4), 120 (day 6), 168 (day 8), 240 (day 11), 288 (day 13), 336 (day 15) h after the start of infusion. The primary plasma PK parameters for isavuconazole were area under the concentration–time curve (AUC) from time of dosing to 72 h (AUC_72_) and maximum concentration (*C*_max_) for the healthy control group compared with participants with ESRD, AUC from time of dosing extrapolated to infinity (AUC_∞_), AUC from time of dosing to last measurable plasma concentration (AUC_last_), and *C*_max_ for the healthy control group compared with participants with mild, moderate, or severe RI. Additional PK parameters for isavuconazole included time to reach *C*_max_ (*t*_max_), total clearance (CL), half-life (*t*_½_), and volume of distribution (*V*_z_). PK parameters for BAL8728 included: healthy, ESRD, and RI participants (day 1): AUC_∞_, AUC_72_, AUC_last_, *C*_max_, *t*_max_, *t*_1/2_, *V*_z_, and CL: ESRD participants (day 15): AUC_72_, *C*_max_, and *t*_max_.

Urine was collected for all able subjects over the following time intervals: day 1 pre-dose (−2 to 0) 0–6, 6–12, 12–24, 24–48, and 48–72 h after start of infusion. PK parameters included the amount and percentage of drug excreted unchanged in the urine (Ae_last_/Ae_72_, for the dialysis comparisons) for all participants and renal clearance (CL_R_ calculated as Ae_last_/AUC_last_) at day 1 for the healthy control group and participants with mild, moderate, or severe RI; dialysis clearance (CL_D_) at day 15 was also assessed for participants with ESRD.

Plasma PK parameters were calculated using WinNonlin^®^ version 5.2 or higher (Certara, Princeton, NJ, USA).

### Safety assessments

Treatment-emergent adverse events (TEAEs; defined as adverse events that started any time after the first dose of study drug was administered through the follow-up visit) were assessed for all participants. The number and percentage of participants with TEAEs were summarized for each renal function group by system organ class.

### Statistical analysis

A sample size of 16 participants (8 per group) in part 1 of the study and 32 participants (8 per group) in part 2 of the study was determined based on the precedent set by other PK studies similar in design. No formal sample size calculation was performed.

The PK analyses used two approaches: One approach compared PK between each renal impaired group (CG method CL_cr_), and a second approach compared the relationship between PK and eGFR (MDRD method). Descriptive statistics (number of participants, mean, and standard deviation, minimum, median, and maximum) were used to summarize continuous variables. Descriptive statistics used for categorical variables consisted of frequency and percentage of participants in each category. In addition, for PK parameters, geometric mean and coefficient of variation were also determined.

To assess the effect of RI on the PK of isavuconazole and BAL8728, an analysis of covariance (ANCOVA) was performed on natural log-transformed AUC_∞_, AUC_last_, and *C*_max_ with renal function group (mild, moderate, or severe RI and healthy control group) as a fixed effect and age, sex, and current smoking status as covariates. The effect of time of dialysis relative to dosing on the PK of isavuconazole and BAL8728 was assessed using ANCOVA on the natural log-transformed AUC_72_ and *C*_max_ between day 1 and day 15 (calculated using pre-dialysis access line concentrations), while subjects were on dialysis from the ESRD group with the visit as a fixed effect (day 1 and day 15), the subject as a random effect, and weight on day 1 and day 15 as a covariate. The 90% confidence intervals (CIs) around the geometric least square mean (LSM) ratios (day 15/day 1) of AUC_72_ and *C*_max_ were constructed.

Covariates were assessed at the 0.1 significance level and removed from the model if insignificant. The 90% CIs around the geometric LSM ratios (severe/healthy control group, moderate/healthy control group, and mild/healthy control group) of AUC_∞_, AUC_last_, and *C*_max_ were constructed. No effect of RI on PK was declared if the corresponding CIs for the ratio fell completely within the interval (70%, 143%) for all three parameters of isavuconazole.

Safety data were analyzed using descriptive statistics. All statistical analyses were performed using SAS^®^ version 9.1 or higher (Statistical Analysis Software, Cary, NC, USA).

## Results

### Patient characteristics

A total of 20 participants were enrolled, and 19 completed part 1 of the study; 29 participants were enrolled and completed part 2 of the study (Table 1). Only five participants were enrolled in the severe RI group due to slow recruitment.

**Table 1:** Demographics and characteristics of participants

	Study part 1		Study part 2			
	Healthy control group (*n* = 9)	ESRD (*n* = 11)	Healthy control group (*n* = 8)	Mild RI (*n* = 8)	Moderate RI (*n* = 8)	Severe RI (*n* = 5)
Age [years], median (range)	48 (19–64)	52 (20–64)	51 (34–57)	63 (51–65)	56 (34–61)	59 (50–62)
Males, *n* (%)	5 (55.6)	5 (45.5)	5 (62.5)	5 (62.5)	3 (37.5)	5 (100)
Race, *n* (%)						
White	8 (88.9)	1 (9.1)	6 (75.0)	8 (100)	4 (50.0)	3 (60.0)
Black or African American	0	10 (90.9)	2 (25.0)	0	3 (37.5)	2 (40.0)
Other	1 (11.1)	0	0	0	1 (12.5)	0
Ethnicity, *n* (%)						
Not Hispanic or Latino	9 (100)	11 (100)	4 (50.0)	3 (37.5)	5 (62.5)	3 (60.0)
eGFR-CG, mean ± SD^a^ eGFR-MDRD, mean ± SD^b^	104.1 ± 18.6 90.7 ± 9.1	8.7 ± 2.3 6.6 ± 1.8	104.6 ± 17.9 94.7 ± 19.8	67.7 ± 7.7 64.1 ± 12.4	39.9 ± 5.7 32.1 ± 5.9	18.5 ± 5.4 14.8 ± 5.2

*CG* Cockcroft–Gault method, *eGFR* estimated glomerular filtration rate, *ESRD* end-stage renal disease, *MDRD* modification of diet in renal disease, *RI* renal impairment, *SD* standard deviation

^a^eGFR-CG (mL/min/1.73 m^2^) × Body surface area/1.73

^b^eGFR-MDRD (mL/min/1.73 m^2^)

### Dialysis and PK of isavuconazole and BAL8728

**Fig. 1:**
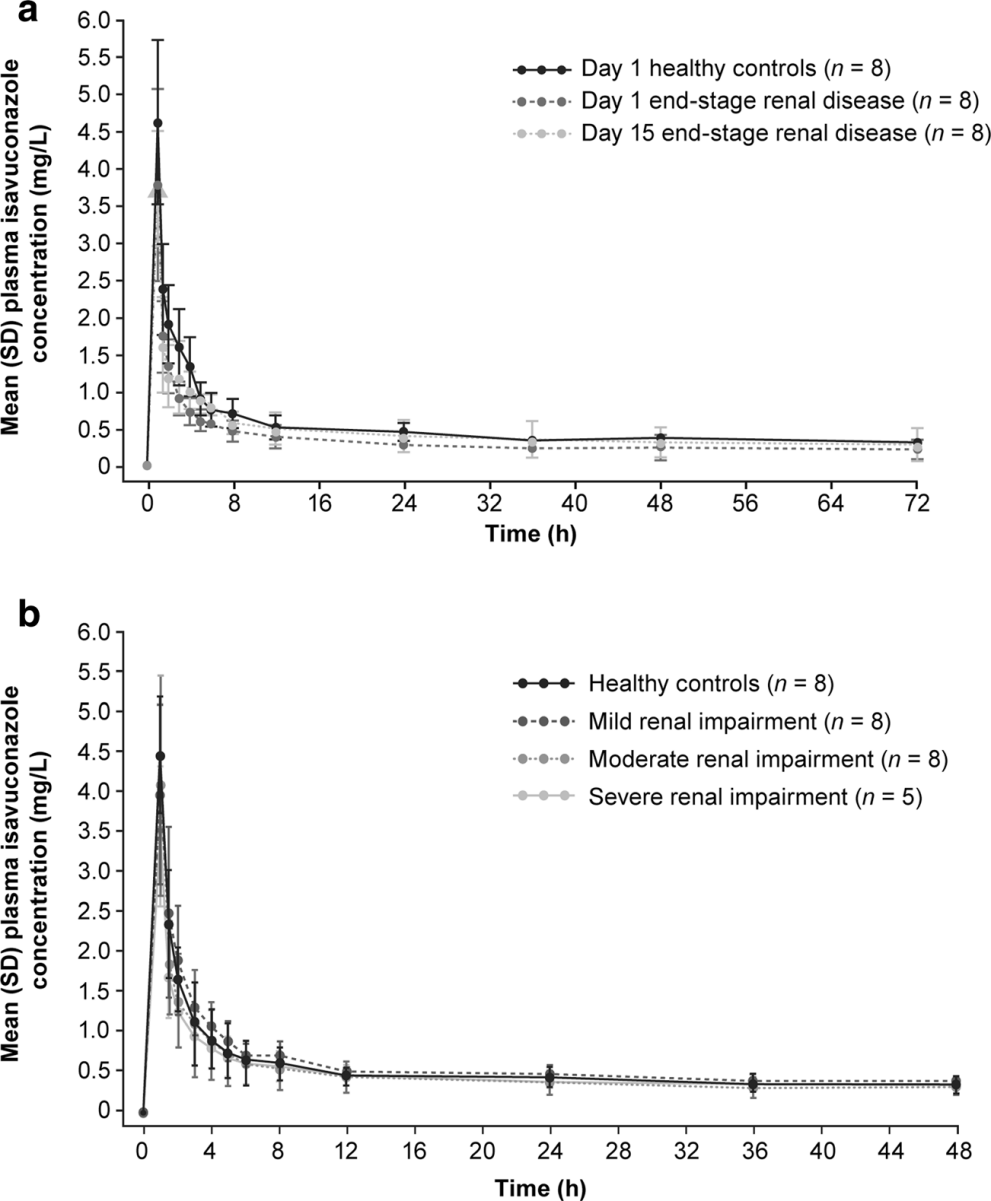
Mean (standard deviation [SD]) plasma concentration–time profiles for isavuconazole in **a** healthy control group (day 1) versus participants with end-stage renal disease on both day 1 and day 15 and **b** for participants with mild, moderate, and severe RI versus the healthy control group

Mean plasma concentration–time profiles for isavuconazole in participants with ESRD compared with the healthy control group are shown in Fig. 1. More than 99.9% of isavuconazole was bound to protein in samples from all treatment groups. On day 1, when isavuconazonium sulfate was administered as a 1-h IV infusion post-hemodialysis in ESRD participants, there was a 34% decrease in AUC_72_ of isavuconazole and a 21% decrease in *C*_max_ compared with the healthy control group dosed under similar conditions (Table 2). BAL8728 *C*_max_ values were 2% lower in participants with ESRD, compared with the healthy control group. The AUC_72_ for isavuconazole increased by 30% and the AUC_72_ for BAL8728 decreased by 22% (Table 2) with dosing of isavuconazole prior to dialysis in participants with ESRD. The day 15 result was similar to the AUC_72_ results obtained for the healthy control group on day 1 (Table 3). The mean *t*_1/2_ of total isavuconazole was approximately 1.6-fold longer in participants with ESRD versus the healthy control group (Table 3). Less than 1% of the administered isavuconazole was recovered in dialysate fluid, consistent with the low dialysis clearance (CL_D_) of 292 mL/h.

**Table 2:** Geometric LSM ratios for isavuconazole and BAL8728 in patients with ESRD versus healthy controls and for ESRD at day 15 versus day 1

Parameter	Ratio study group/study group	Geometric LSM ratio %	90% CI
Isavuconazole			
AUC_72_ *C*_max_	ESRD/healthy controls ESRD/healthy controls	66.3 79.3	50.8–86.7 60.6–103.9
AUC_72_	ESRD day 15/ESRD day 1	130.5	122.8–138.6
BAL8728			
AUC_72_ *C*_max_	ESRD/healthy controls ESRD/healthy controls	110.3 97.7	85.4–142.4 76.0–125.7
AUC_72_	ESRD day 15/ESRD day 1	78.2	68.6–89.0

*AUC*_*72*_ area under the concentration curve at 72 h, CI confidence intervals, *C*_*max*_ maximum plasma concentration, *ESRD* end-stage renal disease, *LSM* least square mean

**Table 3:** Isavuconazole and BAL8728 pharmacokinetic parameters for participants with ESRD and healthy control group

Parameter	Isavuconazole			BAL8728		
	Healthy control group (*n* = 8)^a^	ESRD day 1 (*n* = 8)^b^	ESRD day 15 (*n* = 8)	Healthy control group (*n* = 8)^a^	ESRD day 1 (*n* = 8)^b^	ESRD day 15 (*n* = 8)
AUC_72_, mg*h/L	36.9 ± 9.5	25.1 ± 10.0	32.3 ± 15.4	1.2 ± 0.4	1.3 ± 0.3	0.9 ± 0.2
*C*_max_, mg/L	4.6 ± 1.1	3.7 ± 1.3	3.7 ± 0.8	0.9 ± 0.2	0.9 ± 0.3	0.9 ± 0.2
AUC_∞_, mg*h/L	94.7 ± 32.3	95.7 ± 78.6	–	1.2 ± 0.4	1.3 ± 0.3	–
AUC_last_, mg*h/L	77.9 ± 22.1	62.0 ± 40.2	–	1.1 ± 0.3	1.2 ± 0.3	–
*t*_max_, h	1.0 (1.0–1.1)	1.0 (1.0–1.0)	1.0 (1.0–1.0)	1.0 (1.0–1.1)	1.0 (1.0–1.0)	1.0 (1.0–1.0)
*t*_½_, h	125.5 ± 63.3	204.5 ± 82.6	–	1.3 ± 0.1	1.5 ± 0.3	–
*V*_z_, L	386.2 ± 150.5	735.6 ± 277.3	–	133.3 ± 35.2	144.3 ± 64.3	–
CL, L/h	2.4 ± 0.8	2.9 ± 1.3	–	70.5 ± 23.0	64.5 ± 23.9	–
Ae_last_, %	0.5 ± 0.2	–	–	–	–	–
CL_R_, mL/h	12.5 ± 5.5	–	–	–	–	–
CL_D_, mL/h	–	291.7 ± 87.4	–	–	–	–

All PK data expressed as mean ± standard deviation, except *t*_max_, which is expressed as median (range)

*Ae*_*last*_ cumulative amount of unchanged isavuconazole excreted in the urine, *AUC* area under the concentration–time curve, *AUC*_*72*_ AUC from time of dosing until 72 h, *AUC*_∞_ AUC extrapolated to infinity, *AUC*_*last*_ AUC to last measurable plasma concentration, *C*_*max*_ maximum concentration of isavuconazole, *CL* total clearance of isavuconazole, *CL*_*D*_ dialysis clearance of isavuconazole, *CL*_*R*_ renal clearance of isavuconazole from plasma, *ESRD* end-stage renal disease, *t*_*max*_ time to reach maximum concentration, *t*_*½*_ half-life of isavuconazole

^a^One participant discontinued on day 1

^b^Pharmacokinetic results for three participants with ESRD were unavailable due to a handling error during sample collection resulting in the contamination of *C*_max_ values

### Isavuconazole and BAL8728 PK in renal impairment

There were no consistent changes in *t*_1/2_ of isavuconazole or BAL8728 plasma concentrations observed in participants with mild-to-severe RI versus healthy control group (Table 4; Supplementary Table S1). Compared with the healthy control group, plasma BAL8728 AUC_72_ for subjects with ESRD was 10% higher, whereas the plasma AUC_∞_ in mild, moderate, or severe RI groups were 29, 4, and 24% higher, respectively. A_e_ % and CL_R_ for both isavuconazole and BAL8728 decreased with increasing RI (mild to severe). BAL8728 *C*_max_ values in participants with mild and severe RI were 16 and 11% higher, respectively, and 3% lower in participants with moderate RI.

**Table 4:** Isavuconazole pharmacokinetic parameters for day 1 for healthy participants compared with individuals with renal impairment

Parameter	Healthy control group (*n* = 8)	Mild RI (*n* = 8)	Moderate RI (*n* = 8)	Severe RI (*n* = 5)
AUC_∞_, mg*h/L	98.8 ± 50.5	96.2 ± 46.9	97.2 ± 26.3	98.8 ± 53.9
AUC_last_, mg*h/L	75.8 ± 22.9	77.0 ± 22.8	74.0 ± 20.1	73.6 ± 19.9
*t*_max_, h	1.0 (1.0–1.0)	1.0 (1.0–1.0)	1.0 (1.0–1.0)	1.0 (1.0–1.0)
*t*_½_, h	140.5 ± 77.7	117.0 ± 66.2	158.5 ± 56.4	145.8 ± 65.8
*C*_max_, mg/L	4.4 ± 0.7	3.9 ± 1.1	4.1 ± 1.4	3.4 ± 0.9
CL, L/h	2.4 ± 1.2	2.5 ± 1.0	2.2 ± 0.8	2.4 ± 0.8
Ae_last_, %	0.4 ± 0.2	0.2 ± 0.1	0.1 ± 0.1	0.1 ± 0.03
CL_R_, mL/h	14.0 ± 13.3	6.8 ± 4.3	3.4 ± 2.7	2.0 ± 0.9
*V*_z_, L	410.8 ± 89.7	341.6 ± 72.3	509.1 ± 262.2	439.4 ± 65.4

All data are expressed as mean ± standard deviation, except *t*_max_ which is expressed as median (range)

*Ae*_*last*_ cumulative amount of unchanged isavuconazole excreted in the urine, *AUC* area under the concentration–time curve, *AUC*_*72*_ AUC from time of dosing until 72 h, *AUC*_*∞*_ AUC extrapolated to infinity, *AUC*_*last*_ AUC to last measurable plasma concentration, *CL* total clearance of isavuconazole, *CL*_*R*_ renal clearance of isavuconazole from plasma, *RI* renal impairment, *t*_*max*_ time to reach maximum concentration, *t*_*½*_ half-life of isavuconazole, *V*_*z*_ volume of distribution

**Fig. 2:**
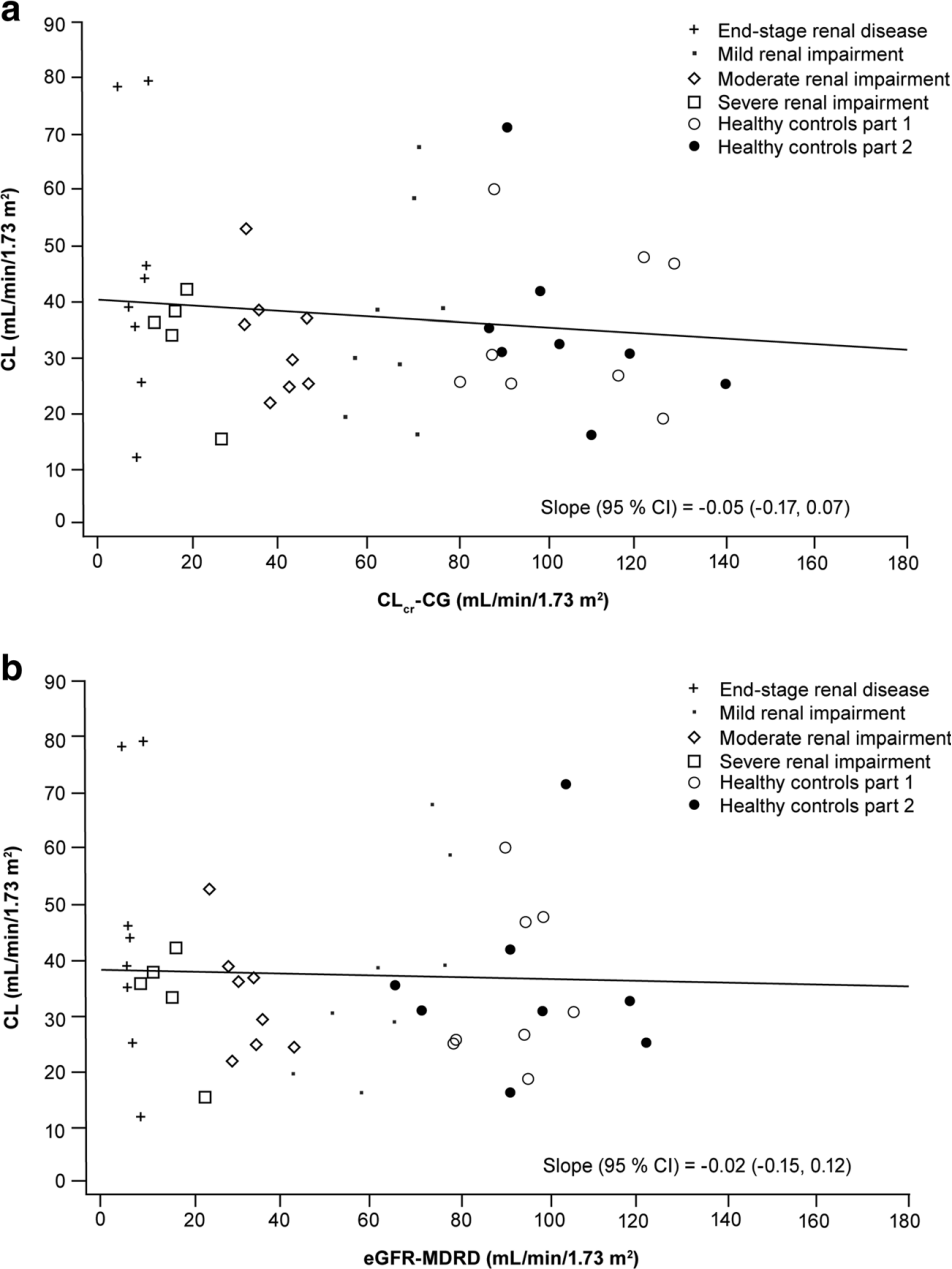
The relationship between total clearance of isavuconazole (CL) and renal function in relation to creatinine clearance (CL_cr_) by Cockcroft–Gault (CG) method (**a**) and estimated glomerular filtration rate (eGFR) by the Modification of Diet in Renal Disease (MDRD) method (**b**). *CI* as confidence intervals

There was no significant relationship between total plasma isavuconazole PK parameters (*C*_max_ and CL) with continuous markers of renal function CL_cr_ and eGFR using either the CG or the abbreviated MDRD formula (Fig. 2, Supplementary Fig. S1). No correlation was identified between BAL8728 PK parameters and markers of renal function (data not shown).

### Urinary excretion

Isavuconazole urinary clearance decreased in parallel with a decrease in renal function (Supplementary Table S2). The amount of isavuconazole excreted unchanged in urine samples was 0.07% of the total dose in patients with severe RI compared with 0.44% in the healthy control group. The small volume of the dialysis clearance in participants with ESRD was consistent with the highly albumin-bound nature of isavuconazole (Supplementary Table S2). BAL8728 was not detected in dialysate samples.

### Safety

Most TEAEs were considered mild. No participant experienced a TEAE that was considered severe, and no deaths were reported during the course of the study. However, one healthy participant in part 1 of the study experienced a TEAE (chest discomfort) during IV administration of isavuconazole that was considered related to the study drug and led to discontinuation from the study (Table 5).

**Table 5:** Summary of treatment-emergent adverse events^a^

Parameter *n* (%)	Study part 1		Study part 2			
	Healthy control group (*n* = 9)	ESRD (*n* = 11)	Healthy control group (*n* = 8)	Mild RI (*n* = 8)	Moderate RI (*n* = 8)	Severe RI (*n* = 5)
TEAEs	7 (77.8)	7 (63.6)	4 (50.0)	5 (62.5)	5 (62.5)	4 (80.0)
Drug-related TEAEs	7 (77.8)	7 (63.6)	4 (50.0)	4 (50.0)	5 (62.5)	3 (60.0)
TEAEs leading to study discontinuation	1 (11.1)^b^	0	0	0	0	0
Most common TEAEs^c^						
General disorders and administration site conditions	6 (66.7)	4 (36.4)	2 (25.0)	2 (25.0)	3 (37.5)	3 (60.0)
Nervous system disorders	3 (33.3)	4 (36.4)	2 (25.0)	2 (25.0)	4 (50.0)	1 (20.0)
Gastrointestinal disorders	0	3 (27.3)	1 (12.5)	1 (12.5)	2 (25.0)	2 (40.0)
Infections and infestations	0	2 (18.2)	0	0	0	1 (20.0)
Skin and subcutaneous tissue	0	2 (18.2)	1 (12.5)	0	0	0

*ESRD* end-stage renal disease, *MedDRA* medical dictionary for regulatory activities, *RI* renal impairment, *TEAE* treatment-emergent adverse event

^a^By MedDRA version 12.1 system organ class

^b^TEAE was considered to be drug-related in this patient

^c^TEAEs occurring in ≥2 patients overall

## Discussion

This study showed that there was no significant impact of renal function measured by either CL_cr_ or eGFR on isavuconazole AUC and *C*_max_ values. The AUC_∞_ and AUC_last_ of plasma isavuconazole in participants with mild, moderate, or severe RI were not significantly different compared with healthy participants with normal renal function. The PK parameters of isavuconazole in plasma were similar between healthy participants with normal renal function and participants with mild, moderate, or severe RI.

Accurate assessment of kidney function is essential for determining appropriate drug dosing regimens [20]. Therefore, eGFR-MDRD equations have been developed to more accurately assess renal function and renal impairment and appropriate drug dosage adjustments [20]. Historically, the CG equation was the method most commonly used to assess drug dosage adjustments in renally impaired patients in clinical practice [20]. Currently, eGFR using the MDRD approach is an alternative approach to the CG method to determine drug dosages in patients with renal impairment [20–22]. However, previous studies have shown discordance rates of up to 40% and significant difference in drug dosing regimens between the MDRD and CG methods [21, 22]. We found no significant relationship between either total plasma isavuconazole *C*_max_ or total body clearance from plasma and renal function assessed by CL_cr_ (CG) or eGFR (MDRD). This is consistent with a population PK study which showed that eGFR used as a covariate did not have a significant effect on clearance of isavuconazole [23]. These findings add further support that dose adjustments of isavuconazole are unlikely to be required in individuals with RI or in those with ESRD who are receiving hemodialysis.

Two approaches were used in the analysis of data: The first approach examined renal function by categorically grouping the severity of impairment (mild, moderate, severe or ESRD as defined by CL_cr_ by CG), and the second approach examined renal function as a continuous variable (eGFR or CL_cr_) related to PK parameters to the measure of renal function. Grouping by severity of renal impairment parallels the clinical approach found in national and international guidelines and is relevant to clinicians familiar with these guidelines [24–29]. The continuous variable approach was objective and independent of empirical classification. Consistency between the two approaches adds robustness to the findings of this study and provides support to its conclusions.

In participants with ESRD, the decrease in AUC and *C*_max_ and wide variability for each when dialysis preceded drug dosing were influenced by intercompartmental fluid shifts intrinsic to hemodialysis and post-dialysis recovery. The clearance of drugs by conventional hemodialysis is predominantly a passive diffusional process driven by unbound concentration gradient between plasma water and dialysate [8, 30]. As the binding of drug to plasma proteins increases, removal of drug by dialysis will decrease [31]. Therefore, hemodialysis did not clear isavuconazole from the plasma of individuals with ESRD due to the high protein binding of isavuconazole (>99.9%) predominantly to albumin. However, the longer half-life and volume of distribution of isavuconazole in individuals with ESRD may also be due to decreased plasma binding by albumin due to uremia which may impact drug metabolism by the liver [32]. The increase in AUC in dialysis patients may be due to the displacement of isavuconazole from albumin by heparin while patients are on dialysis which has been reported for some other drugs [33]. In view of the low and intermittent dialytic clearance of isavuconazole from plasma, it can be concluded that clearance of isavuconazole by thrice weekly dialysis is unlikely to have any appreciable effects on the PK of isavuconazole in ESRD patients. Therefore, post-dialysis supplementation of isavuconazole is unlikely to be required. Conversely, if isavuconazole is inadvertently overdosed, the overdose cannot be effectively managed by hemodialysis.

Analysis of isavuconazole PK across renal function as a continuous variable showed no significant impact of renal function measured by eGFR on *C*_max_ and AUC. Although differences in the renal excretion of isavuconazole were observed among groups with differing levels of renal impairment, the overall level of renal excretion was quite small and the observed differences would not be expected to impact on the PK of isavuconazole in any significant way.

In this study, a single IV infusion of isavuconazole was generally well tolerated by individuals with normal renal function; those with mild, moderate, or severe RI; and those with ESRD. The number and percentage of participants experiencing TEAEs were low and generally similar between groups, and most TEAEs were considered mild. However, more individuals in the ESRD and severe RI groups experienced gastrointestinal disorders compared with those with normal renal function. However, for those receiving hemodialysis, consideration should be given to administering isavuconazole pre-dialysis. Based on the findings of this study, dose adjustments of isavuconazole are unlikely to be required in individuals with RI or in those with ESRD who are receiving hemodialysis.

## Electronic supplementary material

Table S1 (DOC 33 kb)

Table S2 (DOC 31 kb)

Fig. S1 The relationship between maximum concentration (C_max_) of isavuconazole and creatinine clearance (CL_cr_) by the Cockcroft Gault (CG) method (**a**) and estimated Glomerular Filtration Rate (eGFR) by the Modification of Diet in Renal Disease (**b**) (GIF 51 kb)

High Resolution Image (TIFF 1961 kb)
