# Supplementary material for: Phase I trial to investigate the effect of renal impairment on isavuconazole pharmacokinetics
Source: Eur J Clin Pharmacol. 2017 Mar 7;73(6):669–78. doi: 10.1007/s00228-017-2213-7 (PMC5423998; doi:10.1007/s00228-017-2213-7)
Supplement: Supplementary file 1 — (DOC 33 kb) [file 228_2017_2213_MOESM1_ESM.doc]

**Supplementary Table 1** BAL8728 pharmacokinetic parameters for Day 1 for healthy participants compared with individuals with renal impairment

| **Parameter** | **Healthy control group**  **(*n* = 8)** | **Mild RI**  **(*n* = 8)** | **Moderate RI**  **(*n* = 8)** | **Severe RI**  **(*n* = 5)** |
| --- | --- | --- | --- | --- |
| AUC_∞_, mg* h/L | 1.1 ± 0.3 | 1.2 ± 0.2 | 1.1 ± 0.3 | 1.0 ± 0.1 |
| AUC_last_, mg* h/L | 1.1 ± 0.3 | 1.1 ± 0.2 | 1.1 ± 0.3 | 1.0 ± 0.1 |
| t_max_, h | 1.0 (1.0–1.0) | 1.0 (1.0–1.0) | 1.0 (1.0–1.0) | 1.0 (1.0–1.0) |
| t_½_, h | 1.2 ± 0.3 | 1.5 ± 0.5 | 1.4 ± 0.2 | 1.7 ± 0.6 |
| C_max_, mg/L | 0.9 ± 0.2 | 0.9 ± 0.2 | 0.9 ± 0.2 | 0.8 ± 0.06 |
| CL, L/h | 72.1 ± 19.3 | 68.1 ± 15.9 | 69.4 ± 17.8 | 72.9 ± 8.5 |

All data are expressed as mean ± standard deviation, except t_max_ which is expressed as median (range).

Ae_last_, cumulative amount of unchanged isavuconazole excreted in the urine; AUC, area under the concentration-time curve; AUC_72_, AUC from time of dosing until 72 hours; AUC_∞_, AUC extrapolated to infinity; AUC_last_, AUC to last measurable plasma concentration; CL, total clearance of isavuconazole; CL_R_, renal clearance of isavuconazole from plasma; RI, renal impairment; t_max_, time to reach maximum concentration; t_½_, half-life of isavuconazole
