# Supplementary material for: Phase I trial to investigate the effect of renal impairment on isavuconazole pharmacokinetics
Source: Eur J Clin Pharmacol. 2017 Mar 7;73(6):669–78. doi: 10.1007/s00228-017-2213-7 (PMC5423998; doi:10.1007/s00228-017-2213-7)
Supplement: Supplementary file 2 — (DOC 31 kb) [file 228_2017_2213_MOESM2_ESM.doc]

**Supplementary Table 2** Urinary excretion of isavuconazole

|  | **Part 1** | |  | **Part 2** | | |
| --- | --- | --- | --- | --- | --- | --- |
| **Parameter** | **Healthy Control Group (*n* = 9)** | **ESRD   (*n* = 11)** | **Healthy Control Group (*n* = 8)** | **Mild RI   (*n* = 8)** | **Moderate RI  (*n* = 8)** | **Severe RI   (*n* = 5)** |
| Ae_last_ (μg) | 935.2 ± 361.8 | — | 872.6 ± 445.3 | 449.0 ± 198.1 | 255.6 ± 274.3 | 141.7 ± 50.8 |
| CL_R_ (mL/min/1.73 m^2^) | — | — | 0.2 ± 0.2 | 0.1 ± 0.1 | 0.05 ± 0.04 | 0.03 ± 0.01 |
| CL_D_ (mL/h) | — | 291.7 ± 87.4 | — | — | — | — |

Ae_last_, cumulative amount of unchanged isavuconazole excreted in the urine; CL_D_, dialysis clearance of isavuconazole; CL_R_, renal clearance of isavuconazole from plasma; ESRD, end stage renal disease; RI, renal impairment
