# Supplementary figures and images for: Phase I trial to investigate the effect of renal impairment on isavuconazole pharmacokinetics
Source: Eur J Clin Pharmacol. 2017 Mar 7;73(6):669–78. doi: 10.1007/s00228-017-2213-7 (PMC5423998; doi:10.1007/s00228-017-2213-7)

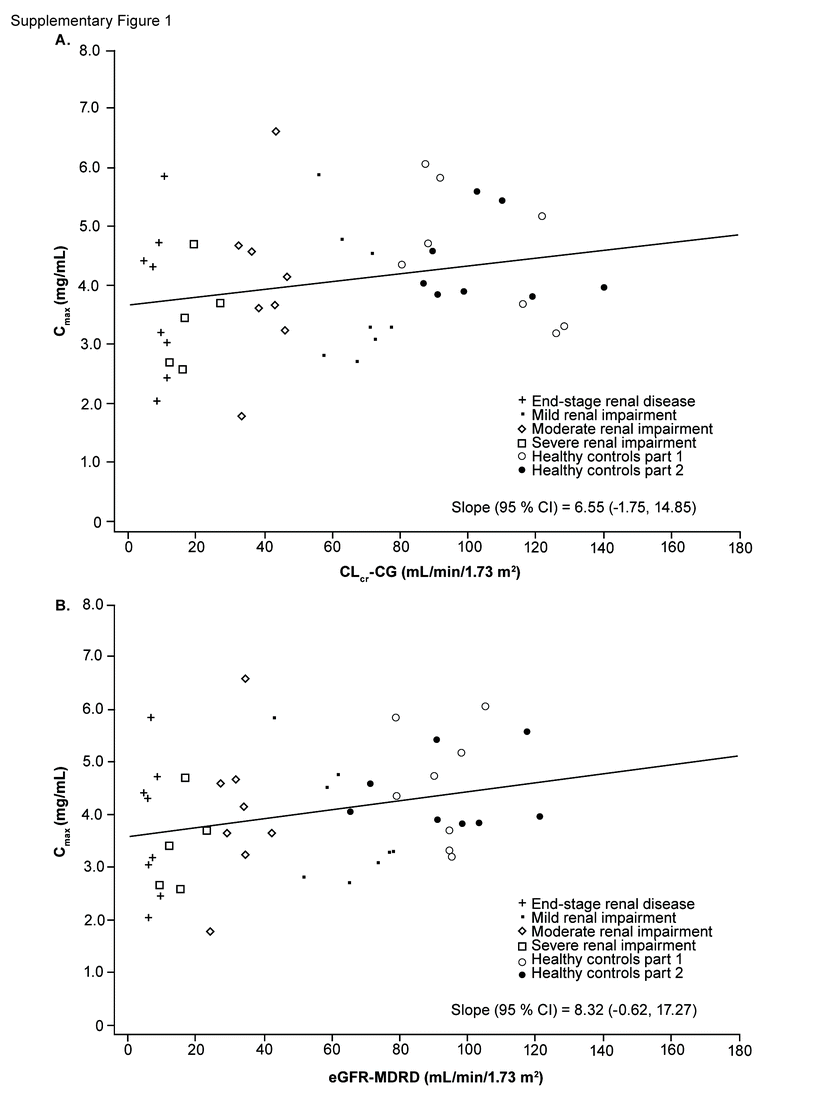

Supplement: Supplementary file 3 — The relationship between maximum concentration (Cmax) of isavuconazole and creatinine clearance (CLcr) by the Cockcroft Gault (CG) method (a) and estimated Glomerular Filtration Rate (eGFR) by the Modification of Diet in Renal Disease (b) (GIF 51 kb) [file 228_2017_2213_Fig3_ESM.gif]

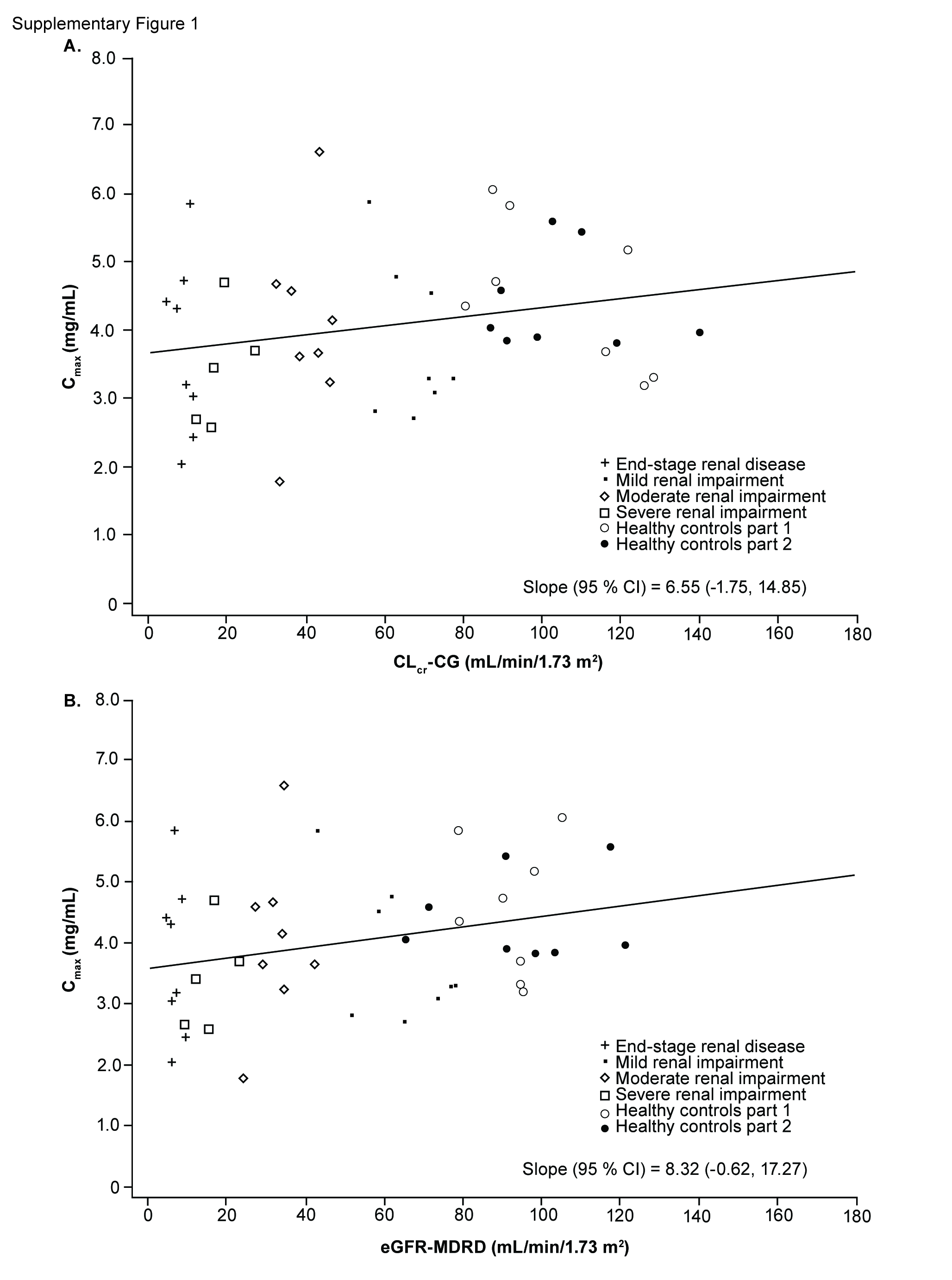

Supplement: Supplementary file 4 — High Resolution Image (TIFF 1961 kb) [file 228_2017_2213_MOESM3_ESM.tif]
